# Novel *ANKRD17* variants implicate synaptic and mitochondrial disruptions in intellectual disability and autism spectrum disorder

**DOI:** 10.1186/s11689-025-09619-3

**Published:** 2025-07-02

**Authors:** Dan Xia, Yuanyuan Xu, Zhanwen He, Rui Chen, Xiaoqin Xiao, Xiaojuan Li, Kewen Deng, Shuyun Deng, Lina Zhang, Jieming Zhang, Xiaofang Peng, Zhe Meng, Ruohao Wu, Dilong Wang, Zulin Liu, Hui Chen, Lu Li, Liyang Liang

**Affiliations:** 1 Cellular and Molecular Diagnostics Center, Sun Yat-Sen Memorial Hospital, Sun Yat-Sen University, Guangzhou, 510220 China; 2 Brain Research Center, Sun Yat-Sen Memorial Hospital, Sun Yat-Sen University, Guangzhou, 510120 China; 3 Department of Children’s Neuro-Endocrinology, Sun Yat-Sen Memorial Hospital, Sun Yat-Sen University, Guangzhou, 510220 China; 4 Department of Obstetrics and Gynaecology, Sun Yat-Sen Memorial Hospital, Sun Yat-Sen University, Guangzhou, 510220 China; 5 Nanhai Translational Innovation Center of Precision Immunology, Sun Yat-Sen Memorial Hospital, Foshan, 528200 China

**Keywords:** *ANKRD17* haploinsufficiency, Intellectual Disability (ID), Autism Spectrum Disorder (ASD), Synaptic protein abnormalities, Mitochondrial inhibition

## Abstract

*ANKRD17* has recently been implicated in intellectual disability (ID) and autism spectrum disorder (ASD); however, the underlying molecular mechanisms remain unclear. Using trio whole-exome sequencing (Trio-WES) and chromosomal microarray analysis (CMA), we identified two unrelated cases with novel de novo heterozygous *ANKRD17* variants. Case 1 describes a fetus with multiple congenital anomalies, where genetic analysis revealed a microdeletion at 4q13.3 truncating the *ANKRD17* gene. Case 2 involves a 12-year-old male presenting with mild ID and progressive social impairments, associated with a NM_032217.5: c.1252 C > T (p.Arg418*) variation in *ANKRD17*. Our study highlighted in mouse models an association between *Ankrd17* haploinsufficiency and deficits in social behavior, spatial learning and memory, as well as elevated anxiety. Furthermore, our studies suggest dysregulation of synaptic proteins and mitochondrial function, along with impaired neural circuits following *Ankrd17* knockdown. These results expand the genetic and phenotypic spectrum of *ANKRD17*-related disorders, underscore the critical role of mitochondrial dysfunction in the pathophysiology of *ANKRD17*-related ID and ASD.

**Supplementary Information:**

The online version contains supplementary material available at 10.1186/s11689-025-09619-3.

## Introduction

*Ankyrin repeat domain 17* (*ANKRD17*) is a gene implicated in intellectual disability (ID) and autism spectrum disorder (ASD). This gene-disease relationship is newly described and considered strong but not yet definitive according to the Clinical Genome Resource (GlinGen, https://curation.clinicalgenome.org/). The primary source of case-level evidence is the study by Chopra et al. [3]. To date, fewer than 40 unrelated individuals with *ANKRD17* variations or microdeletions have been reported worldwide [3, 19, 25, 27, 34]. Haploinsufficiency is strongly suggested as the underlying mechanism of the disease, but functional studies are currently lacking. Therefore, additional case reports and functional research are needed to improve our understanding and treatment of this condition.

*ANKRD17* belongs to a protein family characterized by the presence of ankyrin repeats, which are conserved structural motifs found in various eukaryotic organisms and have diverse biological functions, including transcriptional regulation, cytoskeletal organization, and signal transduction [18]. The gene encodes a protein with two distinctive clusters of ankyrin repeats, a KH domain, and conserved sequences for nuclear export (NES) and nuclear localization (NLS). It is widely expressed across various brain cell types, as indicated by RNA-seq data [3]. In vitro studies suggested that ANKRD17 interacts with cyclin E/CDK2, promoting cell cycle progression [4]. Additionally, *Ankrd17* appears to play a role in immune responses, being involved in antibacterial immunity through NOD1-and NOD2-mediated pathways and antiviral immunity via the RIG-1-like receptor-mediated signaling pathway [20]. The Drosophila counterpart of *ANKRD17*, known as *Mask*, is crucial for tissue growth and functions as a co-factor for the Yorkie transcriptional coactivator, a key component of the Hippo pathway [24]. *Ankrd17* knockout (KO) mice exhibit abnormal vascular maturation, haemorrhage, and embryonic lethality by embryonic day (E) 11 [15], which limits the exploration of the gene’s function. In summary, while some research has explored the diverse functions of *Ankrd17*, the mechanism by which *ANKRD17* may lead to ID and ASD remains largely unexplored.

In this study, we present two cases involving novel *ANKRD17* deficiency. Case 1 describes a fetus with multiple congenital anomalies, where genetic analysis revealed a de novo heterozygous microdeletion at 4q13.3 truncating the *ANKRD17* gene (only exon 1 retained). Case 2 involves a 12-year-old male with mild ID and progressive social impairments, associated with a de novo NM_032217.5: c.1252 C > T (p.Arg418*) variation in *ANKRD17*. Additionally, we performed behavioral experiments using *Ankrd17*-deficiency mice, which mimic impaired social interaction, elevated anxiety, and compromised spatial learning memory. By integrating data from embryonic human brain tissues and mouse models, our findings suggest an association between *ANKRD17* deficiency and disruptions in synaptic proteins and mitochondrial dysfunction.

## Methods and materials

### Patients and animals

The two probands and their respective parents were enrolled in this study at the Sun-Yat sen Memorial Hospital of Sun Yat-sen University. C57BL/6 J mice (SPF grade) were housed and fed at the Medical Research Center Animal Experimental Platform of Sun Yat-sen Memorial Hospital. Male mice aged 4 ~ 5 weeks were used for the experiments.

### Genetic detection

Trio-WES was performed at our Cellular and Molecular Diagnostics Center as previously described [5, 31]. In brief, genomic DNA was isolated from peripheral blood samples preserved in EDTA and obtained from both the probands and their respective parents. The qualified DNAs were further processed using Trio-WES. High-throughput sequencing was conducted on the NovaSeq 6000 platform (Illumina, CA, USA) following DNA library preparation, hybridization capture, enrichment and quality assessment. After filtering and quality control of the raw data, the clean reads were aligned to the University of California Santa Cruz (UCSC) human reference genome (hg19). Several databases were used for variant annotation, including OMIM (http://www.ncbi.nlm.nih.gov/omim/), ClinVar (https://www.ncbi.nlm.nih.gov/clinvar), the Single Nucleotide Polymorphism Database (http://www.ncbi.nlm.nih.gov/SNP/), and gnomAD (http://gnomad-sg.org/). The genetic variations were annotated, analysed, and classified according to the 2015 American College of Medical Genetics (ACMG) guidelines.

### AAV-mediated knockdown of *ANKRD17* in the mPFC and Hippocampus-CA1 region in mice

The adeno-associated virus (AAV) particles used in this study were purchased from Jikai Co. (Shanghai GeneChem, China). Briefly, a series of three *Ankrd17* siRNAs [4] were synthesized, cloned and inserted into the viral vector hSyn promoter-EGFP-MIR155(MCS)-SV40 PolyA (GeneChem, GV680). AAV particles were produced in 293T cells, yielding a final viral concentration of 1.8E + 13 v.g./ml for the recombinant AAV-*Ankrd17* (rAAV-*Ankrd17*). In vivo, the three consecutive *Ankrd17* siRNAs are expected to undergo enzymatic cleavage, generating three separate siRNA molecules. This enhances gene silencing effectiveness, as each siRNA molecule can independently interact with the target RNA, guiding its degradation. An unrelated siRNA duplex provided by Jikai Co. (Shanghai GeneChem, China) was used as a negative control.

To induce *Ankrd17* knockdown in the medial prefrontal cortex (mPFC) or CA1 region of the hippocampus, mice were deeply anaesthetized with isoflurane (approximately 5% in a gas mixture) and injected with either the knockdown or control rAAV virus. The rAAV injection procedure closely followed our previously published methodology [32]. In brief, a total of 300 nanolitres of rAAV-*Ankrd17* virus were bilaterally injected at a rate of 80 nl/min into the mPFC (coordinates: ± 0.35 mm, ML; + 1.8 mm, AP; − 2.4 mm, DV) or the CA1 region (coordinates: −1.9 mm, AP; ± 1.5 mm, ML; − 1.1 mm, DV). Behavioral experiments were conducted three weeks after this surgical procedure.

### Three-chamber social test

Prior to behavioural testing, mice were habituated through gently handling daily for 3–7 days. The standard three-chambered apparatus was used following established methodology [10]. Mice were first acclimated to the apparatus, which included empty cages in both side chambers, for 10 min with free access to all three chambers.

In the sociability stage, a test mouse was placed in the center chamber with doors to the side chambers blocked. One side chamber contained another mouse ("Stranger 1"). After unblocking the doors, the test mouse was allowed to explore the apparatus for 10 min. After a 2-h interval, the social novelty test was conducted. The test mouse was returned to the center chamber, with doors blocked, while a novel mouse ("Stranger 2") was placed in the opposite side chamber. The doors were then unblocked, allowing the test mouse to explore for 3 min.

To eliminate odor cues, the apparatus was cleaned with 75% ethanol between sessions. Video recordings were analyzed for time spent in each chamber, interaction time with each mouse or empty cage (nose proximity ≤ 3 cm), and locomotion patterns using the Smart v3.0 Small Animal Behavioral Recording and Analysis System (Reward Corporation).

### Open-field test

The open-field test was performed in a chamber with dimensions of 50 × 50 × 40 cm (ZH-ZFT, Anhui Zhenghua Biological Instrument Equipment Co., Ltd., Anhui, China). Prior to testing, mice were acclimated to the testing room through handling and familiarization. On the test day, each mouse was individually placed in the center of the chamber and allowed to explore freely for 15 min. Video recordings captured entries into and time spent within the central square and the peripheral areas, as well as the total distance travelled. These data were subsequently quantified in a blinded manner, without knowledge of the experimental groups.

### Y maze

The Y-maze test was conducted in a Y-shaped maze with three arms (A, B, C; 8 × 30 × 15 cm, width × length × height), arranged at 120-degree angles. To maintain cleanliness and eliminate olfactory cues between trials, both the open field and the interior of the Y-maze were thoroughly cleaned with 75% ethanol. The experiment consisted of an exploration period and a testing period. During the exploration period, Arm A was closed. Each mouse was placed at the end of one of the open arms (Arm B), facing the center, and allowed to explore the maze (Arms B and C) for 3 min. After a 2-h interval, all arms of the Y maze were opened, and the mice were placed at the same starting position. During this testing period, the mice were allowed to explore the entire maze for 3 min. For both periods, the number of entries into each arm (A, B, C), the time spent in each arm, and the distance travelled within each arm were recorded for each mouse.

### qPCR

Total RNA was extracted from the mPFC or CA1 region using TRIzol (Thermo Fisher, 15,596,026). RNA concentrations were determined using a NanoDrop ND-2000 (Thermo, United States). To synthesize cDNA, total RNA was reverse transcribed using PrimeScript RT Master Mix (Takara, RR036A) following the manufacturer's instructions. Quantitative Real-time polymerase chain reaction (qPCR) was performed using TB Green™ Premix Ex TaqTM II (Takara, RR820A) on a Roche Real-Time PCR Detection System (LC480). Data were analysed using the 2^−ΔΔCt^ method and normalized to β-actin mRNA levels, which served as an internal control.

The primer sequences are detailed in Table S1, and the *Ankrd17* primers were sourced from previously published literature [15].

### Western blot

The animals were deeply anaesthetized and transcardially perfused with prechilled PBS. Brains were quickly dissected on ice, sliced into 300 μm slices using a Leica Vibratome (VT1200S; Leica, Baden-Baden, Germany) and the target mPFC or CA1 tissues were collected. Tissues were placed in ice-cold RIPA lysis buffer (Sigma, R0278) with a protease inhibitor mixture complete tablet (Sigma, P8340), mechanically homogenized with a pestle, and lysed for 30 min on ice. Lysates were centrifuged at 14,000 rpm for 30 min at 4 °C, and the supernatant was transferred to a fresh tube on ice. Protein concentrations were determined using a BCA assay (Pierce) according to the manufacturer’s protocol. Protein levels were normalized with RIPA lysis buffer, and 5 × sample buffer was added. The lysates were boiled at 95 °C for 10 min for SDS‒PAGE.

Proteins were separated using a premade 4–20% gel (ACE, ET15420) and transferred to 0.22 μm or 0.45 μm PVDF membranes. Membranes were blocked for 2 h in 5% milk prepared in Tris-buffered saline supplemented with 0.05% Tween (TBST). After blocking, membranes were probed overnight at 4 °C with the appropriate primary antibody in TBST. Membranes were washed 3 times in TBST and incubated for 1 h with HRP-conjugated secondary antibody: goat anti-mouse IgG (1:5,000; Cell Signaling Technology, 7076), goat anti-rabbit IgG (1:5,000; Abcam, ab6721)]. After secondary incubation, membranes were washed three times with TBST. Blots were visualized using a chemiluminescence detection system.

Primary antibodies used in this study included: anti-PSD95 (1:1000; Abcam, ab238135), anti-synapsin I (1:1000; Abcam, ab254349), anti-NMDAR2A (1:1000; Abcam, ab133265), Anti-Glutamate Receptor 1(1:1000; Abcam, ab183797,), anti-ANKRD17 (1:1000; Abcam, ab85726; 1:1500; Proteintech, 27701–1-AP), and anti-alpha tubulin (1:1000; Abcam, ab176560).

### Immunofluorescence

Mouse brain tissue was processed as described in our previous publication [32]. Briefly, upon removal, whole brains were fixed overnight in 4% paraformaldehyde at 4 °C, followed by cryoprotection in 30% sucrose for 48 h. Subsequently, the brains were OCT-embedded and sectioned at 30 µm using a cryostat (Leica CM1950, Germany). Sections from the mPFC or CA1 region were selected and stained.

For immunofluorescence, the primary antibodies used were: anti-ANKRD17 (1:2000; Bethyl, IHC-00596), anti-NeuN (1:300; Sigma, MAB377), anti-Camk2a (1:250; Santa Cruz, sc-13141; 1:250; Abcam, ab52476), anti-GAD67 (1:50; Abcam, ab213508), anti-PV (1:1000; Abcam, ab11427,), anti-MAP2 (1:500; Abcam, ab5392) and anti-c-fos (1:250; Santa Cruz Biotechnology, sc-166940). The secondary antibodies were: Alexa Fluor® 488 goat anti-rabbit IgG H&L (1:1000; Invitrogen, ab150077), Alexa Fluor™ 488 goat anti-Chicken IgY (H + L), (1:500; Thermo Fisher Scientific, A-11039) or Alexa Fluor® 647 goat anti-mouse secondary antibody (1:1000; Thermo Fisher Scientific, A21235). Cell nuclei were labelled with DAPI.

Three to four location-matched sections from each mouse were selected, with five mice per group, resulting in a total of 15–20 images per group. To minimize batch effects, samples from the control and knockdown groups were stained together.

### 5D label-free proteomics

Brain tissue was obtained from a 26-week-old fetus with *ANKRD17* deficiency (Case 1), with family consent, due to multiple anomalies identified through ultrasound. The control group included brain tissue from a 25-week-old fetus of the same sex, induced with family consent due to isolated chondrodysplasia detected by ultrasound, with negative WES results. Proteins were extracted from paraffin-embedded brain tissue sections per group for subsequent proteomic analysis.

5D proteomics was conducted by Shanghai Jikai Gene Chem in China. For bioinformatics analysis, Blast2GO (version 1.3.3) was used for GO annotation of the target proteins. During the KEGG pathway annotation process, KOALA (KEGG Orthology and Links Annotation, Version 2.3) software was used to align the KEGG GENES database (version: KO_INFO_END) with the target protein sequences. These protein sequences were classified by KO labels, and pathway information was automatically obtained. Enrichment analysis for both GO annotations and KEGG pathway annotations was performed using Fisher's exact test. WoLF PSORT was subsequently used to predict the subcellular localization of the proteins. Differentially expressed proteins were defined as those with|fold change|≥ 2.

### Statistical analyses

All data were analysed and visualized using R 4.1.1 and Adobe Illustrator 2023. Ordinary two-way ANOVA followed by Bonferroni's multiple comparisons test and two-tailed unpaired *t*-test were employed for comparisons. A *p*-value < 0.05 was considered statistically significance. Data are presented as the mean ± SEM. *, *p* < 0.05; **, *p* < 0.01; ***p < 0.001;****, *p* < 0.0001.

## Results

### Case presentation

#### Case 1

**Fig. 1:**
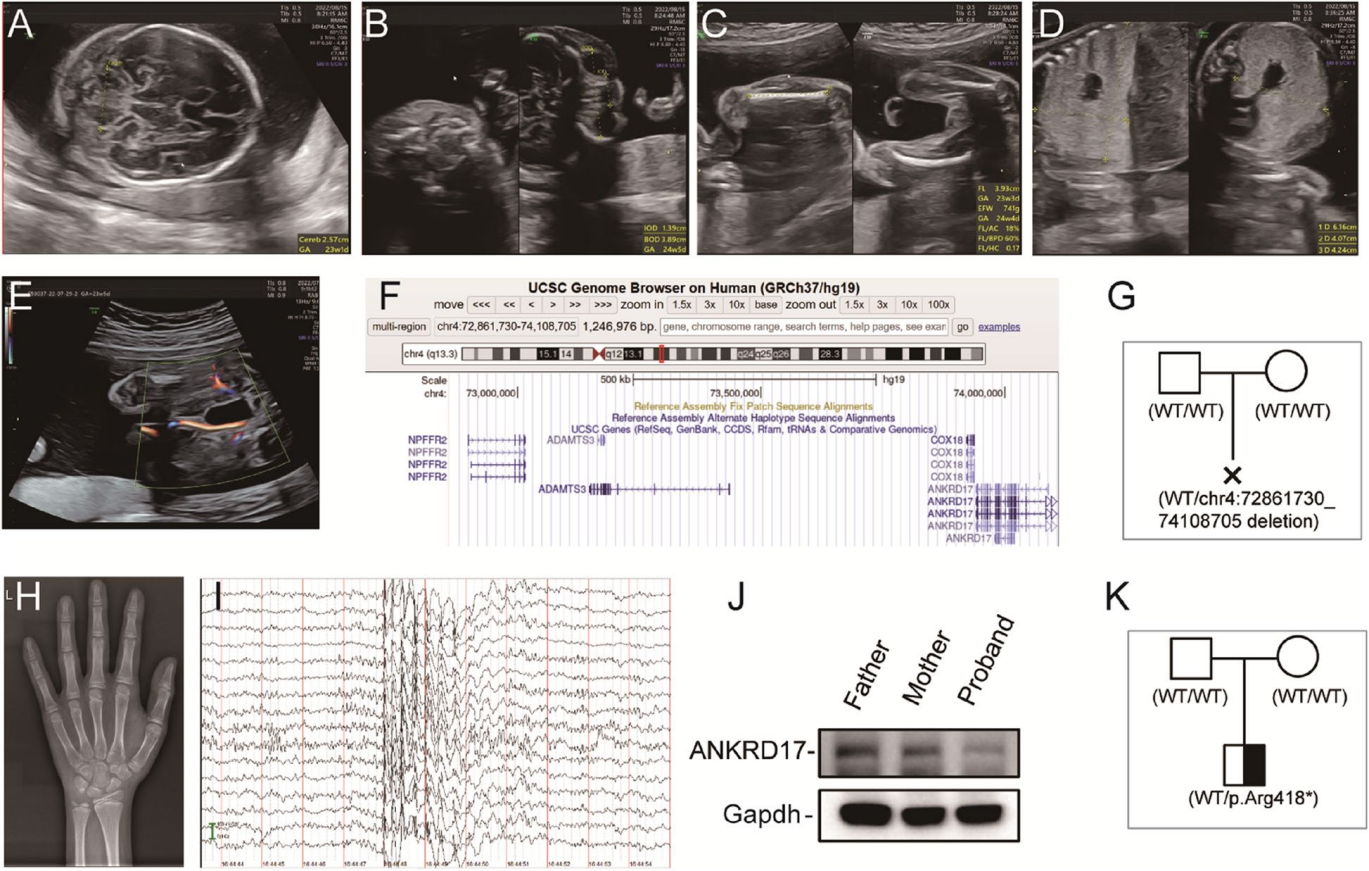
Clinical and genetic characteristics of two unrelated probands with *ANKRD17* deficiency at our hospital. **A**-**E** Ultrasound image of the fetus. **A** Images of the small cerebellum, (**B**) hypertelorism, (**C**) short limbs, (**D**) airway obstruction, (**E**) single umbilical artery. **F** Genomic location and University of California Santa Cruz (UCSC) genes with the deletion observed in this case. **G** Pedigrees of case 1 revealed a de novo deletion. **H** Radiograph of the left hand at the age of 12 years and 7 months of case 2. **I** EEG image showing bilateral synchronous high-amplitude bursts of slow and spiky waves during wake of case 2. **J** Western blot revealed that expression of the ANDKRD17 protein from peripheral blood in the proband 2 was halved compared with that in his parents. **K** Pedigrees of case 2 revealed a de novo nonsense variation

The mother, aged 29, and her partner underwent chromosome karyotype analysis, revealing that the mother carried a translocation between chromosomes 13 and 15, denoted as t (13;15) (q32; q21), while the father had a normal karyotype (46XY). The first-trimester Down syndrome screening revealed a critical-risk result for trisomy 21 (1/270–1/1000) and 18 (1/350–1/1000), based on the combined assessment of nuchal translucency (NT) and maternal serum markers (free β-hCG and PAPP-A). At 19^+^ weeks of gestation, an ultrasound examination revealed abnormal fetal thoracic and abdominal images. Multiple fetal anomalies were observed, including a small cerebellum (transverse diameter < −2 SD; Fig. 1A), widened interocular distance (Fig. 1B), growth retardation (Femur Length [FL] < −2 SD, Humerus Length [HL] < −1.5 SD; Fig. 1C), right airway obstruction (Fig. 1D), a single umbilical artery on the left side (Fig. 1E), and tricuspid regurgitation. Additionally, abnormal fetal soft markers were noted, including a thickened nuchal fold (NF) of 0.63 cm at 23 weeks 0 days, which exceeds the normal threshold of < 0.6 cm for this gestational age.

Trio-WES and CMA identified a de novo heterozygous microdeletion spanning approximately 1.247 Mb at 4q13.3 (chr 4: 72861730_74108705, GRCh37/hg19) (Fig. 1F, G, and S3). This deletion is not documented in the Database of Genomic Variants (DGV, http://dgv.tcag.ca/dgv/app/home) or the Genome Aggregation Database (gnomAD, http://gnomad-old.broadinstitute.org/) and does not overlap with known microdeletion syndromes in the DECIPHER database (https://www.deciphergenomics.org/). The deletion affects four protein-coding genes: three are completely deleted (*NPFFR2*, *ADAMTS3*, *COX18*), while *ANKRD17* is partially truncated with only exon 1 retained (exons 2–34 deleted). Notably, only *ANKRD17* and *ADAMTS3* were identified as morbid genes. *ANKRD17* is associated with autosomal dominant Chopra-Amiel-Gordon syndrome in OMIM, characterized by clinical features, including growth retardation, which align with the phenotype observed here. In contrast, *ADAMTS3* is associated with autosomal recessive Hennekam lymphangiectasia-lymphedema syndrome 3 (OMIM#618154), but the inheritance pattern and phenotype do not match the clinical findings in this case. In addition to these morbid genes, we assessed *NPFFR2* and *COX18*, which are not linked to known diseases at present. *NPFFR2*, involved in pain modulation and energy balance, and *COX18*, essential for mitochondrial cytochrome c oxidase assembly, both exhibit low pLI scores (0.01 and 0.00, respectively), suggesting tolerance to loss-of-function.

#### Case 2

Proband 2, a 12-year-old male born to healthy, non-consanguineous parents, was delivered spontaneously at 41 weeks of gestation. As the first-born of three siblings, his birth parameters were within normal range (weight 3,000 g [0 SD], length 50 cm [0 SD]) with no congenital abnormalities observed. Physical examination at age 12 years showed short stature (height: 131 cm [−3.3 SD], weight: 29.5 kg [−2.1 SD], BMI: 17.2 kg/m^2^ [P25-50]), with suboptimal development and nutritional status. Bone age assessment at 12 years 7 months indicated a delay of 1 year (11 years 6 months) by the Greulich–Pyle method (Fig. 1H).

Electroencephalogram (EEG) revealed paroxysmal bilateral synchronous high-amplitude spikes and slow waves during wakefulness (Fig. 1I), though no seizures were documented. Brain Magnetic Resonance Imaging (MRI) and spine radiography showed no abnormalities. Cardiac ultrasound indicated normal left ventricular systolic function with mild tricuspid and pulmonary valves regurgitation. Abdominal ultrasound (hepatobiliary system, pancreas, kidneys, and urinary tract) was unremarkable. Thyroid function, IGF-1, IGFBP-3, and 25-hydroxyvitamin D levels were within normal limits, while bone-specific alkaline phosphatase (BALP) was elevated at 81 μg/L (reference: 4–21 μg/L).

Developmental delays were noted from 6–7 months of age. He received a diagnosis of mild intellectual disability (Wechsler Intelligence Scale score: 52 at 12.5 years). Behavioral assessment disclosed progressive social impairment since age 7, characterized by peer avoidance, stranger anxiety, and ritualistic behaviors (e.g., rock-building). Physiological fear responses (clenched fists, trembling) and emotional dysregulation (irritability, anxiety/depression) were observed, consistent with comorbid ASD and social anxiety disorder.

Genetic detection was done due to his global developmental delay and short stature with intellectual disability. Trio-WES analysis identified a de novo heterozygous nonsense variant in *ANKRD17* (NM_032217.5: c.1252 C > T(p.Arg418*)) in exon 7/34 (Fig. 1K). Western blotting confirmed 50% reduced ANKRD17 protein expression versus parental controls (Fig. 1J). This novel variant is absent in the Genome Aggregation Database (gnomAD) and has not been reported in the literature.

### *Ankrd17* knockdown in the mPFC induces autism-like phenotypes and impaired spatial memory in mice

**Fig. 2:**
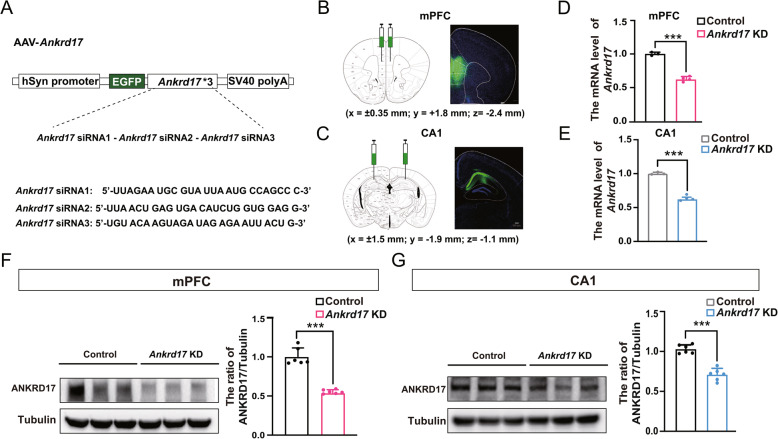
AAV-mediated *Ankrd17* knockdown in the mPFC and hippocampal CA1 region in mice. **A **Schematic representation of the AAV-*Ankrd17* construct, which features an hSyn promoter-driven EGFP sequence followed by three *Ankrd17* siRNAs arranged in a tandem array. **B**, **C** Successful expression of the injected AAV virus (green) in the mPFC (**B**) and CA1 region (**C**), with nuclei visualized by DAPI (blue). Scale bar = 200 μm. **D**, **E** Reductions in mRNA expression levels detected by qPCR in both the mPFC (**D**) and CA1 region (**E**). **F**, **G** Reductions in protein expression levels detected by WB in both the mPFC and CA1 region. KD, knockdown

Three weeks after tandem rAAV viral injection, green fluorescence was observed in the target mPFC and hippocampal CA1 regions (Fig. 2A–C). qPCR and Western blot analyses confirmed successful knockdown of ANKRD17 expression. Specifically, the mRNA levels were reduced to 62.2% (Fig. 2D) and 64.4% (Fig. 2E) of control levels, and the protein levels were decreased to 53.4% (Fig. 2F) and 72.1% (Fig. 2G) of control. These results effectively mimic the heterozygous deficiency of ANKRD17 in mice.

**Fig. 3:**
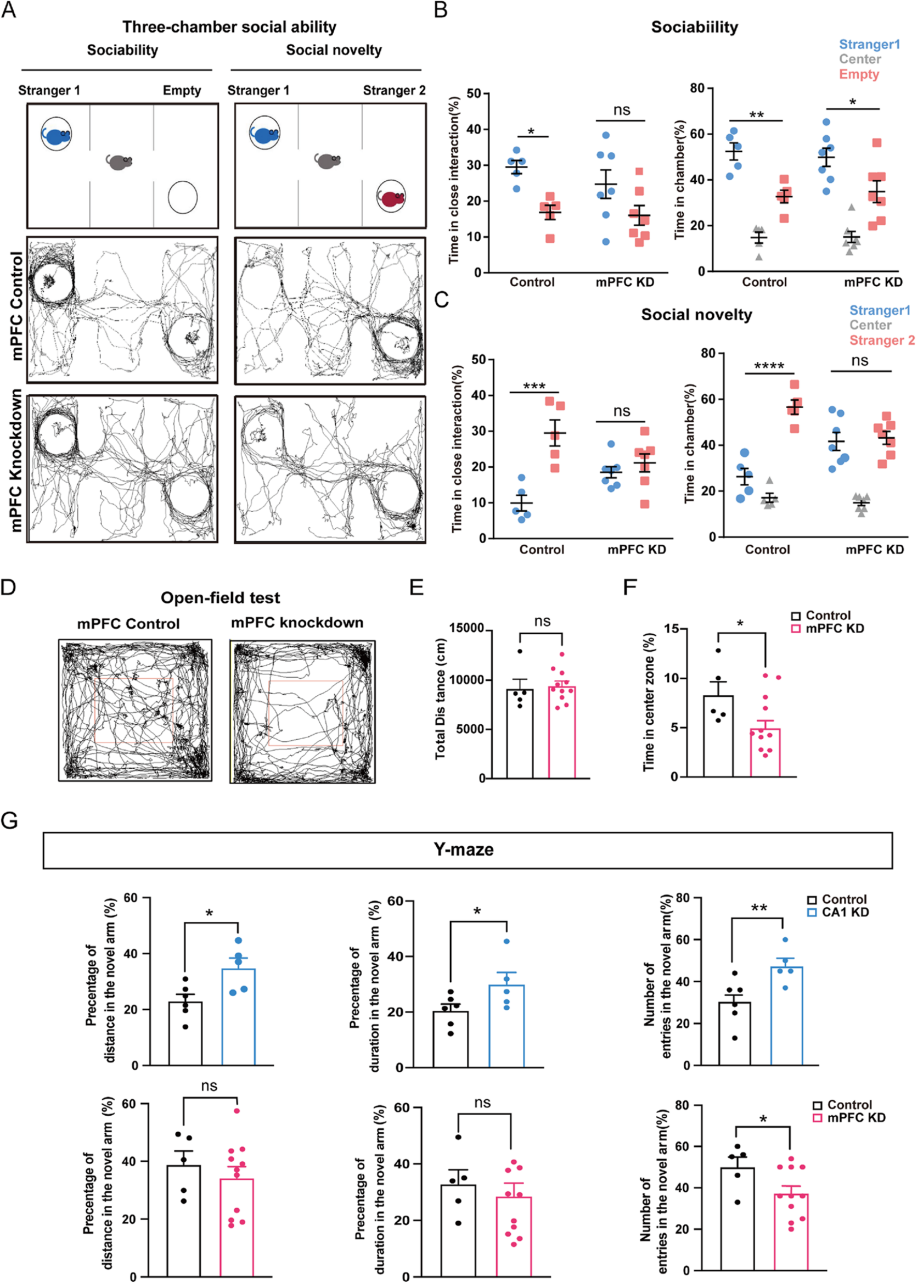
*Ankrd17* knockdown results in autism-like phenotypes and impairs spatial memory in mice. **A** Diagram of the three-chamber test (top) and representative traces of a control mouse (middle) and an *Ankrd17*-knockdown mouse (bottom) during the sociability and social novelty stages. **B** Sociability test: time in close interaction (left) and time in the chamber (right). **C** Social novelty test: time spent in close interaction (left) and time spent in the chamber (right). **D**-**F** Elevated anxiety levels in mPFC-*Ankrd17* knockdown mice. **D** Representative traces of a control mouse (left) and an *Ankrd17*-knockdown mouse (right) in the open-field area, with the red line marking the center zone. **E** Quantification of total distance travelled and (**F**) time spent exploring the centre zone during the open-field test. **G** Comparisons of the percentage of distance travelled, duration of entry, and number of entries into the novel arms between CA1-*Ankrd17* knockdown mice (upper) or mPFC-*Ankrd17* knockdown mice (lower) and their respective controls

To characterize the social behaviour of mPFC-*Ankrd17*-knockdown mice, we used the three-chamber social task (Fig. 3A, top), a well-established test for assessing mouse sociability and interest in social novelty. As expected, control mice spent significantly more time in close interaction with Stranger 1 than near empty cages (Fig. 3A, middle; Fig. 3B, left), demonstrating typical social tendencies. However, *Ankrd17*-knockdown mice did not show a significant increase in the time spent in close contact with Stranger 1 compared to the time spent near empty cages (Fig. 3A, bottom; Fig. 3B, left), indicating compromised social inclination. When analysing the time spent within the chamber, *Ankrd17* knockdown did not significantly impair the social preference of mice towards Stranger 1 (Fig. 3B, right). To assess interest in social novelty, Stranger 2 was introduced (Fig. 3A, top). Unlike control mice, *Ankrd17*-knockdown mice showed no preference for Stranger 2, both in terms of time spent in close interaction (Fig. 3A, bottom; Fig. 3C, left) and time spent in the chamber (Fig. 3C, right).

We then conducted an open field test to evaluate the anxiety levels of mPFC control and *Ankrd17*-knockdown mice. The knockdown mice exhibited no signs of locomotor deficits (Fig. 3D, E), but they displayed a significant reduction in the time spent in the center of the open field (Fig. 3D, F), suggesting an elevated anxiety level.

Considering that patients with heterozygous *ANKRD17* deficiency exhibit varying degrees of ID, we explored whether our knockdown mice displayed learning and memory deficits paralleling human conditions. To assess memory, we employed the Y maze test, which evaluates both spatial and recognition memory. In CA1-*Ankrd17*-knockdown mice, we observed a significant increase in the percentage of distance travelled, duration of movement, and number of entries into the novel arms compared to control mice (Fig. 3G, upper). In contrast, mPFC-*Ankrd17*-knockdown mice showed different results; the percentage of distance travelled and duration in the novel arms did not significantly differ from control mice, while the percentage of entries into the novel arms was significantly lower (Fig. 3G, lower). These findings suggest that spatial and recognition memory are impaired in mice with *Ankrd17* knockdown in the mPFC.

### *Ankrd17* knockdown impairs synaptic protein in neurons

**Fig. 4:**
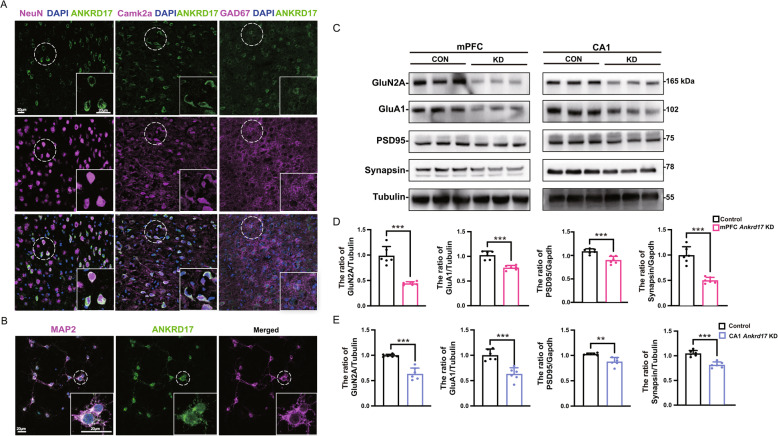
Localization of ANKRD17 expression and impaired synaptic protein composition in *Ankrd17*-deficiency mice. **A** Representative images showing co-staining of ANKRD17 (green) with NeuN, Camk2a, and GAD67 (purple) in mPFC of adult mice. **B** Representative images showing co-staining of ANKRD17 (green) with MAP2 (Purple) in the primary neurons of cortex from late-stage embryos and cultured in vitro for 7 days. Nuclei are stained with DAPI (blue) for cell localization. Bar = 20 μm. **C** Western blotting analysis of synaptic-related proteins in mPFC and CA1 region of control and *Ankrd17*-knockdown mice. **D**-**E** Quantification of protein expression levels in mPFC and CA1. KD, knockdown; CON, control

Information regarding the expression of ANKRD17 in various cell types within mouse brain tissues was previously lacking. To address this, we co-stained ANKRD17 with cell type markers and discovered that ANKRD17 is broadly expressed, particularly in the cytoplasm, of both excitatory and inhibitory neurons in the adult mouse brain (Fig. 4A, S1, and S2). Consistently, in primary neurons derived from the late embryonic mouse cortex, ANKRD17 also showed predominant cytoplasmic localization (Fig. 4B).

Neurodevelopmental disorders, such as autism and intellectual disability, are frequently linked to alterations in synaptic protein composition. To investigate this connection, we performed Western blot analysis of synaptic proteins. Our findings revealed a decrease in the expression of the NMDAR subunit GluN2A, the AMPAR subunit GluA1, the excitatory synaptic scaffolding protein PSD-95 and Synapsin I-a key protein involved in synaptic vesicle storage and release-in both the mPFC and hippocampal CA1 region in *Ankrd17*-knockdown mice compared to those in control mice (Fig. 4C, D, and E).

### Impaired neural circuits in *Ankrd17*-knockdown mice

**Fig. 5:**
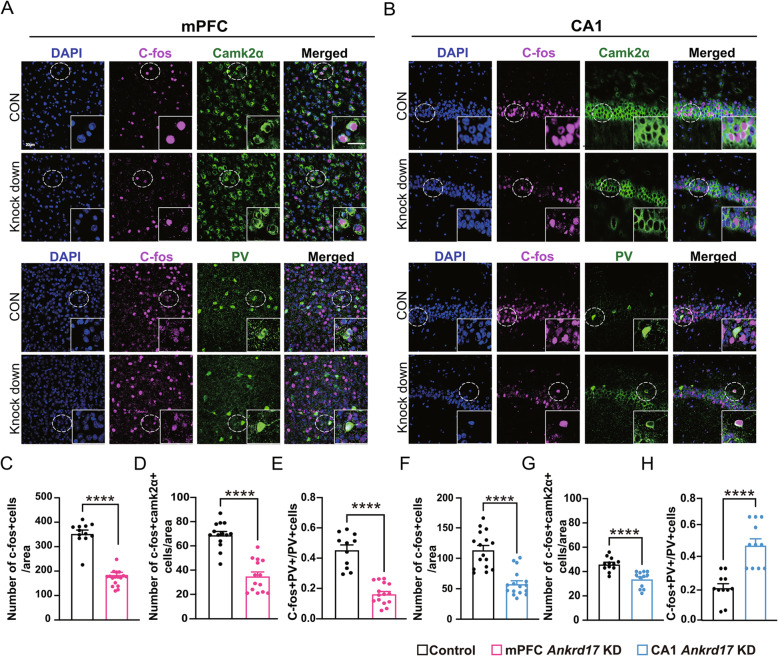
*Ankrd17*-knockdown mouse exhibit deficits in neural circuits. **A** Representative images showing the co-staining of c-fos (purple) with Camk2a (green) or PV (green) in the mPFC and (**B**) in the hippocampal CA1 region. Nuclei were stained with DAPI (blue) for cell localization. Bar = 20 μm. **C-H** Comparisons of c-fos^+^ density, c-fos^+^Camk2a^+^ density, and c-fos^+^PV^+^/PV^+^ between control and *Ankrd17* knockdown mice in the mPFC (**C**-**E)** and in the CA1 region (**F**–**H**). KD, knockdown; CON, control

Our subsequent objective was to investigate potential impairments in neural circuits in *Ankrd17*-knockdown mice. We observed a significant reduction in the density of c-Fos-labelled cells in both the mPFC (Fig. 5A and C) and hippocampal CA1 regions (Fig. 5B and F) in the knockdown mice compared to controls. This reduction suggests that fewer neural circuit cells were activated during behavioral tests in *Ankrd17*-knockdown mice, indicating impaired neural circuits in these brain regions. To further identify the specific types of affected cells, we performed co-staining of c-Fos with Camk2a or parvalbumin (PV) to label excitatory neurons or PV inhibitory neurons, respectively. In both brain regions, co-staining of c-Fos with Camk2a was significantly reduced in the knockdown group (Fig. 5A, B, D and G). Conversely, the proportion of c-Fos^+^ PV^+^ neurons among the PV^+^ neurons was reduced in the mPFC (Fig. 5A and E) but increased in the CA1 region of the hippocampus (Fig. 5B and H).

### Proteomics suggest mitochondrial inhibition in *ANKRD17*-deficient human brain tissue

**Fig. 6:**
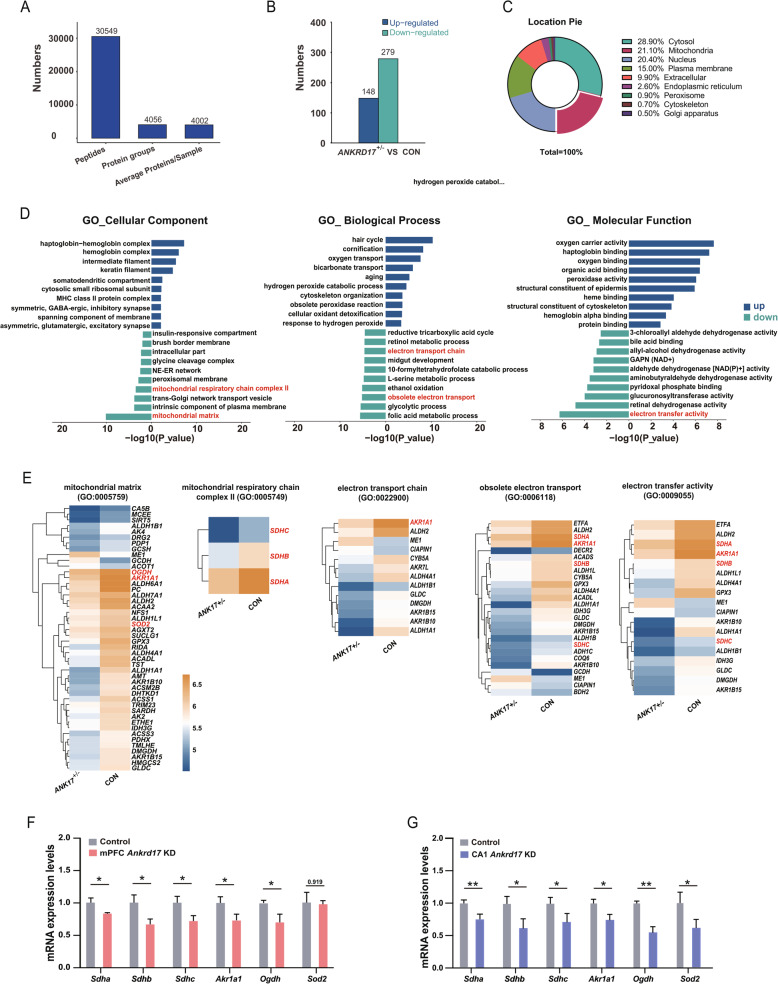
Proteomics suggest mitochondrial inhibition in *ANKRD17*-deficient human brain tissue. **A** Number of peptides and proteins detected by 5D proteomics analysis. **B** Number of up-regulated and down-regulated proteins identified through 5D proteomics. *ANKRD17*^+/-^, cortex brain tissue from case 1 with *ANKRD17* Heterozygous deficiency. CON, cortex brain tissue from a control fetus without *ANKRD17 *deficiency. **C** Proportion of cellular localization of differentially expressed proteins. **D** GO enrichment of up-regulated and down-regulated differentially expressed proteins. GO terms associated with mitochondrial function are highlighted in red. ER-NE network: nuclear outer membrane-endoplasmic reticulum membrane network; GAPN (NAD+): glyceraldehyde-3-phosphate dehydrogenase (NAD+) (non-phosphorylating) activity. **E** Heatmaps showing the expression of down-regulated proteins associated with mitochondrial function, as indicated by the GO terms highlighted in red in panel D. Proteins selected for qPCR verification are marked in red. The heatmap was generated using log10-transformed expression values. **F-G** qPCR verification of genes related to mitochondrial function

To elucidate the molecular differences associated with *ANKRD17* deficiency, we conducted 5D proteomics on postmortem embryonic brain tissues. This comprehensive analysis detected 30,549 peptides and 4,056 proteins (Fig. 6A). Among these, we identified 148 up-regulated and 279 down-regulated differentially expressed proteins (DEPs) in *ANKRD17*-deficiency brain tissues to the non-*ANKRD17*-deficiency brain tissues (Fig. 6B). Notably, 21.1% of the DEPs, the second highest proportion, are located in the mitochondria (cytosol 28.9%, mitochondria 21.1%, nucleus 20.4%, and plasma membrane 15.0%) (Fig. 6C).

We performed GO functional enrichment analyses separately for up-regulated and down-regulated DEPs to determine the functional changes associated with *ANKRD17* deficiency. Interestingly, we found that the down-regulated proteins were significantly enriched in GO terms related to mitochondrial function, such as “mitochondrial matrix” (GO:0005759), “mitochondrial respiratory chain complex II, succinate dehydrogenase complex (ubiquinone)” (GO:0005749), “electron transfer activity” (GO:0009055), obsolete electron transport (GO:0006118), and “electron transport chain” (GO:0022900) (Fig. 6D, E). In contrast, the up-regulated proteins did not show significant enrichment in mitochondrial function-related GO terms (Fig. 6D).

Due to the limited availability of human brain tissue, we selected six mitochondrial function-related genes-*Sdha, Sdhb, Sdhc, Akr1a1, Ogdh,* and *Sod2*-for qPCR verification using mouse brain tissues (Fig. 6E). In both the mPFC and CA1 regions, *Sdha, Sdhb, Sdhc, Akr1a1, and Ogdh* were significantly decreased following *Ankrd17* knockdown, with the exception of *Sod2*, which only decreased in the CA1 region while not in the mPFC (Fig. 6F and G). These results suggest that the gene expression changes observed in the qPCR analysis of mouse brain tissue are generally consistent with the trends of protein changes in embryonic human brain tissue.

Collectively, these findings suggest an inhibition of mitochondria-related functions in embryonic human brain tissue with *ANKRD17* deficiency.

## Discussion

This study integrates clinical cases with knockdown mouse models to confirm the association between *ANKRD17* deficiency and autism, anxiety, and spatial memory impairments. Additionally, our findings suggest disruptions in synaptic protein expression and mitochondrial inhibition, offering novel insights into the molecular roles of *ANKRD17*.

### Clinical implications and phenotypic variability

Previous studies have highlighted the crucial role of the Hippo pathway in brain development, including the cerebellum [26]. Notably, our study is the first to report cerebellar hypoplasia in *ANKRD17*-related disorders, aligning with recent insights into Hippo-YAP signaling: single-cell RNA sequencing data indicate co-expression of *ANKRD17* and *YAP1* in radial glia within the developing human telencephalon [3], and murine studies show *Yap1's* critical role in cerebellar development through regulation of Nestin^+^ glial precursors and Sonic Hedgehog (Shh)-dependent granule cell proliferation [8, 33]. These findings suggest that disruptions in Hippo-YAP signaling may contribute to cerebellar hypoplasia. Further research is essential to elucidate the mechanisms through which YAP1 influences cerebellar growth and function.

Establishing robust genotype–phenotype correlations remains challenging. While deletions involving *ANKRD17*—such as our case 1 (1.247 Mb deletion) and the 1.56 Mb deletion reported by Maldziene et al. [19]—often correlate with severe congenital anomalies, Chopra et al.'s cohort found comparable prenatal abnormalities (9/40 cases) across various variant types (missense, frameshift, and nonsense). Additionally, Yu et al. described a nonsense variant (NM_032217.5:c.1525 C > T(p. Arg 509*)) associated with isolated clubfeet [34], whereas Silverstein et al. reported a missense variant (NM_032217.5: c.7778 A > C (p. His 2593 Pro)) linked to both clubfeet and postnatal neurodevelopmental delays ophthalmologic abnormalities, and dysmorphic craniofacial features, despite the absence of prenatal anomalies [25]. These findings highlight the difficulty of establishing a clear genotype–phenotype correlation and underscore the need for larger cohort studies to identify potential trends.

These cases raise two critical considerations. First, not all congenital anomalies can be detected via prenatal ultrasound. While major structural abnormalities may be identified, neurodevelopmental outcomes—such as developmental delay, ID, and ASD—remain undetectable, despite being major concerns for prospective parents. Second, the identification of prenatal anomalies depends on multiple factors, including fetal positioning and the expertise of the sonographer. As a result, a prenatally diagnosed “isolated” anomaly may not be truly isolated. This underscores the importance of early genetic screening and molecular diagnosis to improve risk assessment and clinical management of *ANKRD17*-related disorders.

### Behavioral implications

Our study explored the behavioral and synaptic implications of *Ankrd17* knockdown in mice, specifically focusing on the hippocampal CA1 region and the mPFC. The results from the Y maze experiments demonstrated distinct impacts on spatial memory and exploratory behavior, providing insight into the roles of these brain regions in cognitive function. In the Y maze test, mice with *Ankrd17* knockdown in the CA1 region exhibited a significant increase in both the number of entries into the novel arm and the time spent exploring it. The CA1 region is critical for encoding and retrieving spatial memory [7, 21], and our findings suggest that reducing the expression of *Ankrd17* in this region of adolescent mice is not sufficient to impair spatial memory function. The increased exploration could be indicative of a compensatory mechanism, where the deficiency in *Ankrd17* leads to an unexpected boost in the ability to navigate and remember new environments.

Conversely, mice with *Ankrd17* knockdown in the mPFC showed a decrease in the number of entries into the novel arm, with no significant change in exploration time within the novel arm. This suggests impairments in decision-making and executive functions, which are primarily governed by the mPFC. The mPFC’s involvement in working memory and decision-making is well-documented, and disruptions in this area can lead to difficulties in retaining and manipulating information about new environments [1]. The differing results in the Y maze experiment following *Ankrd17* knockdown in the CA1 and mPFC regions highlight the distinct functions of these brain areas in spatial memory, decision-making, and emotional regulation. The CA1 region primarily engages in encoding and retrieving spatial memory, while the mPFC is crucial for decision-making, executive functions, and emotional regulation. Moreover, the mPFC exhibits a high degree of plasticity throughout life, even in adulthood [17]. Additionally, the observed decrease in entries into the novel arm might reflect increased anxiety levels as observed in the mPFC-*Ankrd17* knockdown mice, affecting their willingness to explore new environments [2, 14].

### Molecular mechanisms and mitochondrial dysfunction

Our proteomic analysis of embryonic human brain tissue suggested significant down-regulation of mitochondrial function-related proteins in the context of *ANKRD17* deficiency. Specifically, proteins associated with the mitochondrial matrix, respiratory chain complex II, and electron transfer were notably reduced. This suggests that *ANKRD17* deficiency may lead to mitochondrial dysfunction, which could contribute to the neurological deficits observed in patients. Given the high energy demands associated with the frequent firing activity, neurons are particularly sensitive to disruptions in mitochondrial function. Mitochondrial dysfunction has been implicated in various neurological disorders that often co-occur with ID, such as ASD and attention deficit hyperactivity disorder (ADHD) [6, 11, 22, 23]. This suggests a common underlying mechanism involving mitochondrial function. Several ways in which mitochondrial dysfunction may contributes to these disorders, such as oxidative stress [29, 30], altered synaptic function [28, 29], disrupted neurodevelopment [22, 28], impaired neuroplasticity [30], and metabolic disturbances [13]. Additionally, as mitochondria are vulnerable to environmental factors, the result makes the mitochondrion potentially a crucial mediator of genetic-environmental interactions [9], which may also underling the differences in clinical characteristics among individuals. In reality, mitochondrial dysfunction seems prevalent in 5% to 80% of ASD children [12, 23]. A recent clinical trial investigated the use of suramin, a medication that affects cellular metabolism, including mitochondrial function, in children with ASD. The trial suggested that suramin might help alleviate some ASD symptoms by improving mitochondrial function and reducing oxidative stress, thus offering a potential therapeutic avenue [16].

However, due to the rarity of the disease and the challenges in obtaining brain tissue samples, our study involved only one brain tissue sample from a case with *ANKRD17* deficiency. The qPCR validation in mouse brain tissues further supports these findings, showing decreased expression of mitochondrial function-related genes, such as *Sdha, Sdhb, Sdhc, Akr1a1, and Ogdh* in both the mPFC and CA1 regions following *Ankrd17* knockdown. This alignment between human proteomic data and mouse genetic studies strengthens the evidence for mitochondrial impairment as a key mechanism in *ANKRD17*-related disorders. In summary, our findings underscore the importance of mitochondrial function in the pathophysiology of *ANKRD17*-related disorders and highlight potential therapeutic targets for intervention. Future studies are needed to investigate how *ANKRD17* regulates mitochondrial and synaptic functions in detail.

### Brief conclusion

Collectively, our study expands the spectrum of *ANKRD17*-related disorders to include cerebellar hypoplasia and bronchial dysplasia. By integrating clinical cases with knockdown mouse models, we demonstrate that ANKRD17 haploinsufficiency is associated with deficits in social behavior and increased anxiety. Our findings suggest dysregulation of mitochondrial function, which could contribute to the disorder’s pathophysiology. Overall, our combined human and mouse data provide preliminary yet critical insights into the genetic and phenotypic underpinnings of *ANKRD17*-related disorders.

## Supplementary Information

Supplementary Material 1.

## Data Availability

No datasets were generated or analysed during the current study.
