# Supplementary material for: Novel ANKRD17 variants implicate synaptic and mitochondrial disruptions in intellectual disability and autism spectrum disorder
Source: J Neurodev Disord. 2025 Jul 2;17:36. doi: 10.1186/s11689-025-09619-3 (PMC12219137; doi:10.1186/s11689-025-09619-3)
Supplement: Supplementary file 1 — Supplementary Material 1. [file 11689_2025_9619_MOESM1_ESM.docx]

- Supplementary Figures for Western blotting

Figure 1J


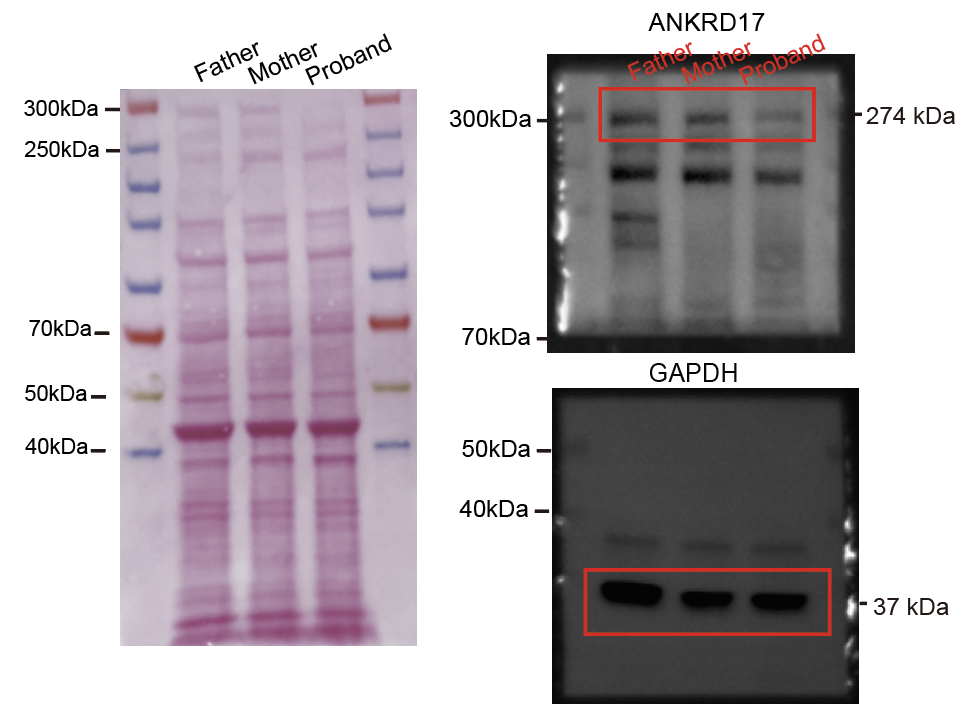


*The band inside the red box is the target band

Figure 2 and 4


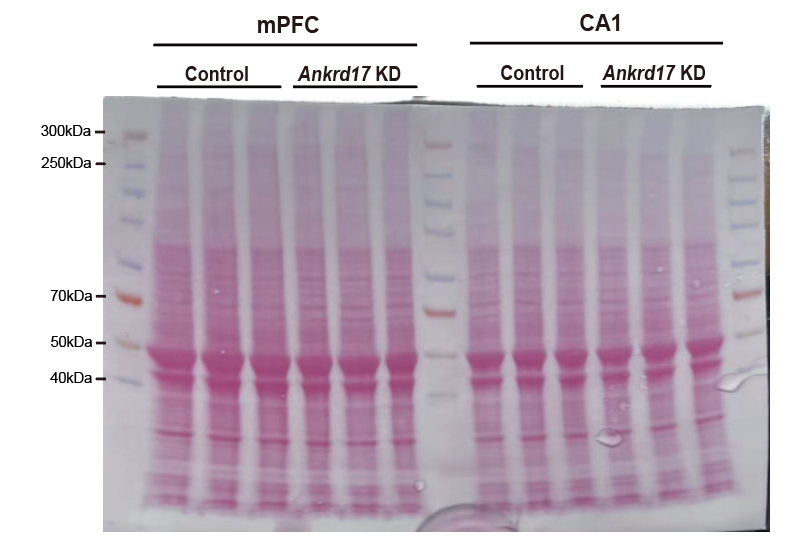


Figure 2F


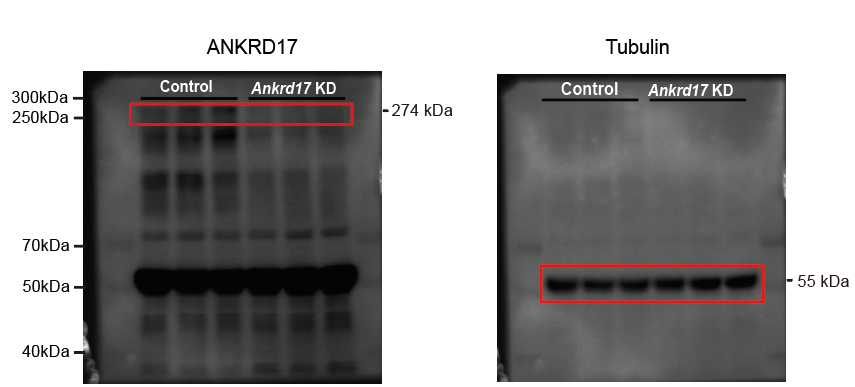


Figure 2G


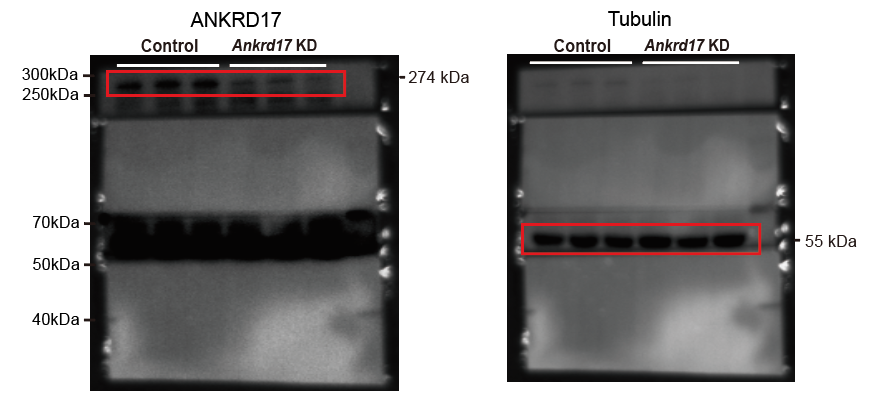


Figure 4C


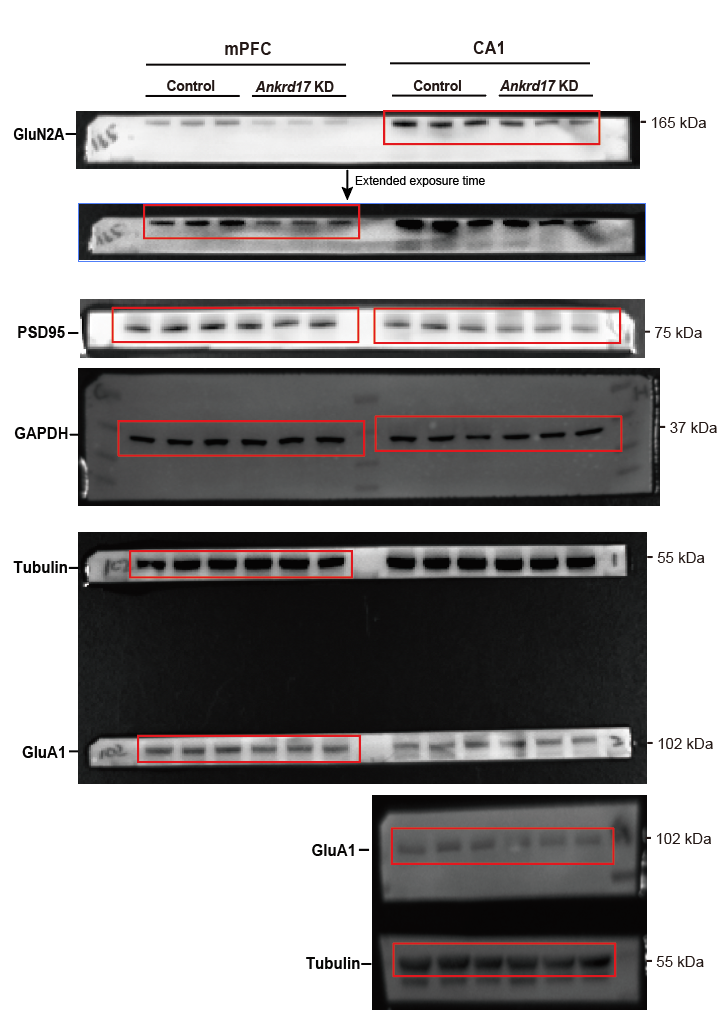


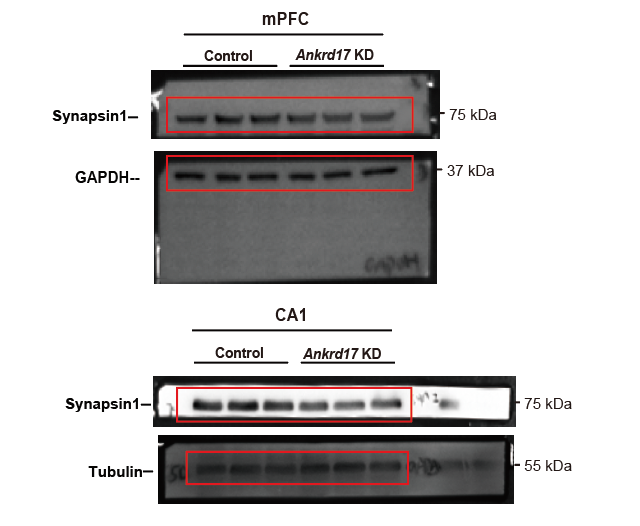


- Supplementary Figures of Subcellular localization of ANKRD17 in neurons


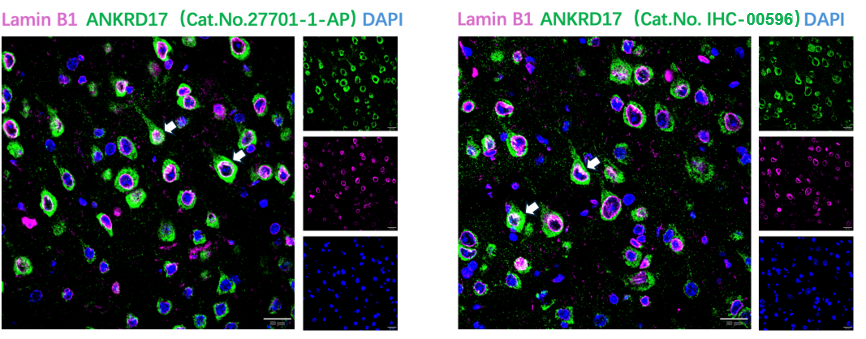


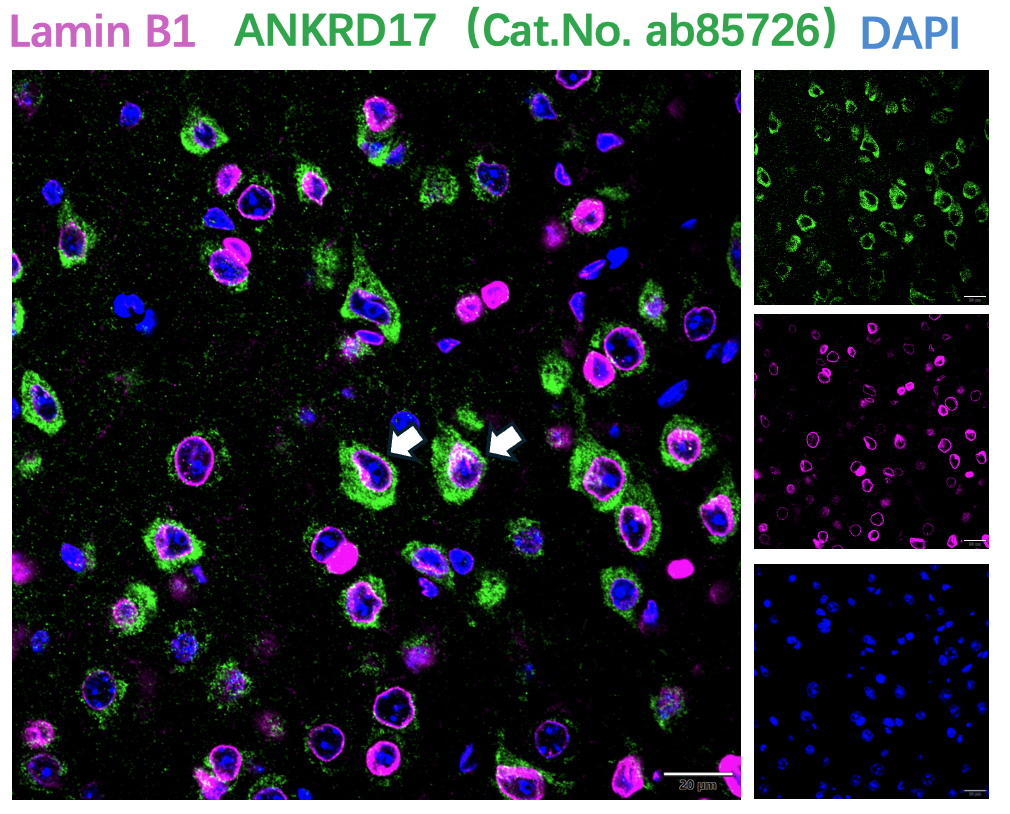


**Figure S1. Immunofluorescence (IF) of ANKRD17 in adult mouse brains using different commercial antibodies.**


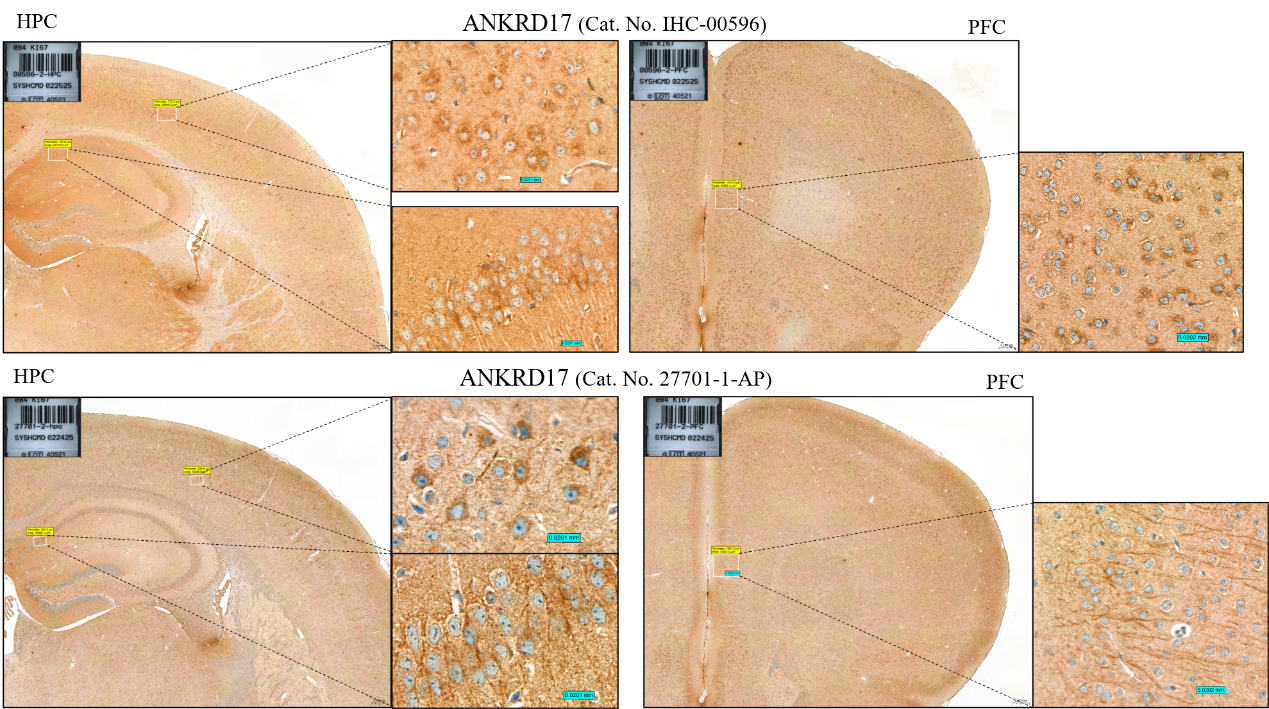


**Figure S2. Immunohistochemistry (IHC) of ANKRD17 in adult mouse brains using different commercial antibodies. HPC:** **Hippocampus; PFC:** **Prefrontal Cortex.**

- Confirmation of Trio-WES-Detected Variants in Cases 1 and 2.

Case 1


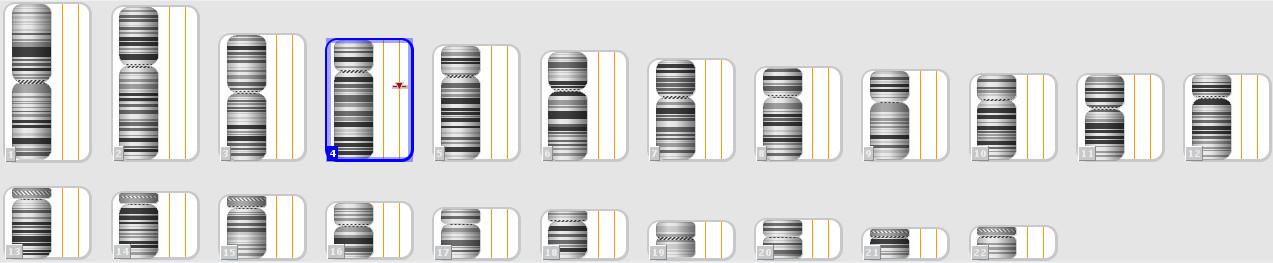


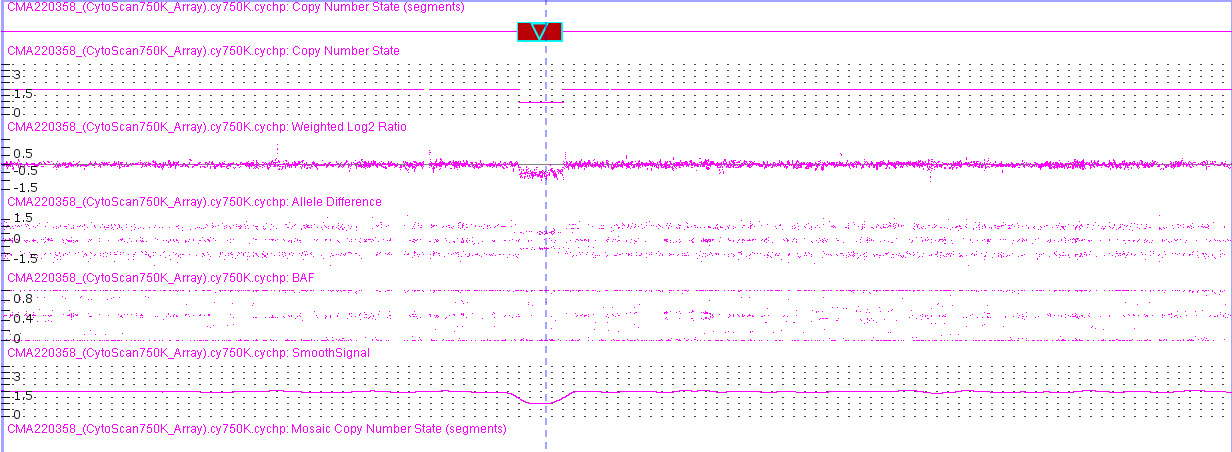


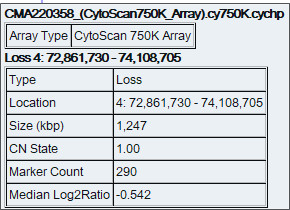


**Figure S3. Chromosomal microarray confirmation.** For Case 1, the copy number deletion was confirmed by chromosomal microarray (CMA) analysis (750K array) of amniotic fluid, with maternal DNA contamination testing performed to ensure reliability. The heterozygous microdeletion spanning approximately 1.247 Mb at 4q13.3 (chr 4: 72861730_74108705, GRCh37/hg19) was show by the ChAS (Chromosome Analysis Suite) analysis software accompanying Affymetrix.

Case 2


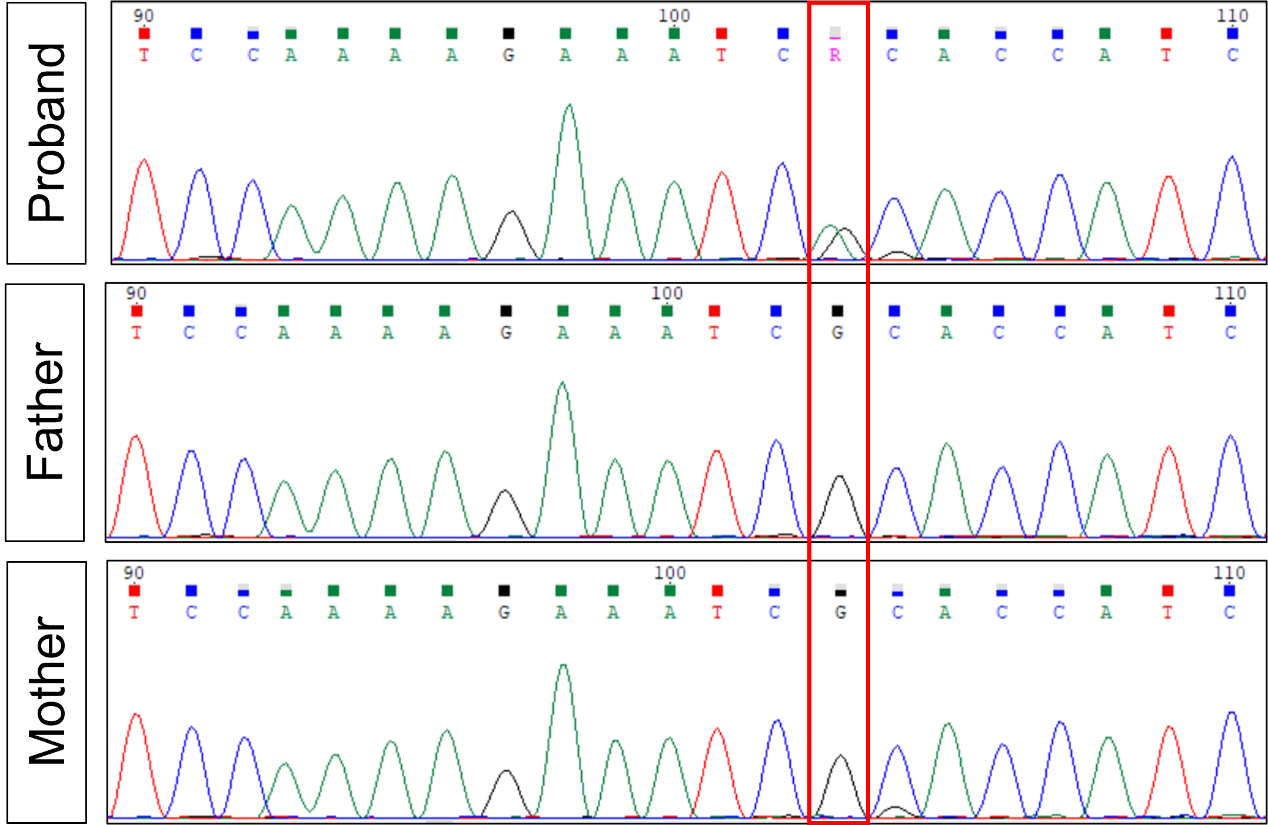


**Figure S4. Sanger sequencing.** For Case 2, the variant (NM_032217.5: c.1252C>T: (p.Arg418*)) was validated via Sanger sequencing.

**Table S1. Primer sequences used in qPCR.**

| **Genes** | **5’to 3’** | **Primer sequence** |
| --- | --- | --- |
| *Ankrd17* | Forward | GCTGATTCATTTGAGTCACCA |
|  | Reverse | CATTTCTTCATGTCCTTCACGAG |
| *β-actin* | Forward | CATGTACGTTGCTATCCAGGC |
|  | Reverse | CTCCTTAATGTCACGCACGAT |
| *Sdhb* | Forward | ATTTACCGATGGGACCCAGAC |
|  | Reverse | GTCCGCACTTATTCAGATCCAC |
| *Sdha* | Forward | GGAACACTCCAAAAACAGACCT |
|  | Reverse | CCACCACTGGGTATTGAGTAGAA |
| *Sdhc* | Forward | GCTGCGTTCTTGCTGAGACA |
|  | Reverse | ATCTCCTCCTTAGCTGTGGTT |
| *Sod2* | Forward | CAGACCTGCCTTACGACTATGG |
|  | Reverse | CTCGGTGGCGTTGAGATTGTT |
| *Ogdh* | Forward | GTTTCTTCAAACGTGGGGTTCT |
|  | Reverse | GCATGATTCCAGGGGTCTCAAA |
| *Akr1a1* | Forward | AGCCTGGTCAGGTGAAAGC |
|  | Reverse | GGCCTCCCCAATCTCAGTT |
| *Scgn* | Forward | GACAACGCACGCAGAAAAACT |
|  | Reverse | TCCTCAGTGCCAGATTTTGCC |
| *Sptbn4* | Forward | CTCGACGCTAACCGCAAGT |
|  | Reverse | CTGGGTCTGGAGCTTGAAGA |
